# Metabolic Responses of Grapevine Leaves to Grapevine Leafroll-Associated Virus 3 Infection

**DOI:** 10.3390/metabo16060359

**Published:** 2026-05-27

**Authors:** Ivana Tomaz, Darko Preiner, Nina Buljević, Darko Vončina

**Affiliations:** 1 Department of Viticulture and Enology, Faculty of Agriculture, University of Zagreb, Svetošimunska Cesta 25, 10000 Zagreb, Croatia; dpreiner@agr.hr; 2 Centre of Excellence for Biodiversity and Molecular Plant Breeding, Faculty of Agriculture, University of Zagreb, 10000 Zagreb, Croatia; nbuljevic@agr.hr; 3 Department of Plant Pathology, Faculty of Agriculture, University of Zagreb, Svetošimunska Cesta 25, 10000 Zagreb, Croatia

**Keywords:** grapevine leafroll-associated virus 3, phenolic compounds, volatile organic compounds, oxidative stress

## Abstract

**Background**: Grapevine leafroll-associated virus 3 (GLRaV-3) is a severe phloem-limited grapevine virus, meaning it is restricted to sugar-transporting vascular tissue, which helps explain its strong effects on leaf physiology and carbon transport. However, its impact on leaf oxidative status, phenolic composition, and volatile organic compounds (VOCs) remains insufficiently characterized. **Methods**: Virus-free and GLRaV-3-infected grapevine leaves were analyzed for photosynthetic pigments, oxidative stress markers, phenolic compounds, and VOCs using spectrophotometric assays, HPLC-DAD/FLD, and SPME-Arrow-GC/MS. Data were evaluated using one-way ANOVA, multiple testing correction, principal component analysis (PCA), and exploratory partial least squares-discriminant analysis (PLS-DA). **Results**: GLRaV-3-infected leaves showed lower chlorophyll a (576.75 vs. 657.85 mg 100 g^−1^ DW), chlorophyll b (282.96 vs. 314.05 mg 100 g^−1^ DW), and total carotenoids (125.89 vs. 154.65 mg 100 g^−1^ DW), but higher malondialdehyde (11.91 vs. 8.73 nmol g^−1^ DW), H_2_O_2_ (0.36 vs. 0.25 μmol g^−1^ DW), and proline (8.83 vs. 7.98 μmol g^−1^ DW). Phenolic profiling showed increased levels of several flavonols and hydroxycinnamic acids, including kaempferol-3-O-glucuronide (2.81-fold), myricetin-3-O-glucoside (1.75-fold), quercetin-3-O-glucuronide (1.48-fold), and caffeic acid (1.30-fold). VOC profiling revealed higher relative abundances of several green leaf volatile-related compounds and methyl salicylate, including 1-methoxy-2-propanol (1.85-fold), 1-penten-3-ol (1.58-fold), hexanal (1.42-fold), and methyl salicylate (1.37-fold). PCA summarized treatment-related differences, with the first two components explaining 63.73% of phenolic and 74.09% of VOC variability, while exploratory PLS-DA/VIP analysis further supported the identification of treatment-associated discriminant metabolic features. **Conclusions**: GLRaV-3 infection is associated with reduced pigment content, increased oxidative stress markers, and coordinated changes in phenolic and VOC profiles. These metabolite changes provide insight into grapevine responses to viral infection and highlight GLRaV-3-associated metabolic features for future targeted studies of grapevine leafroll disease.

## 1. Introduction

Grapevine leafroll disease (GLD) is one of the most widespread and severe viral diseases of grapevine, causing yield losses, delayed ripening, and reduced fruit and wine quality in many viticultural regions worldwide [1,2,3]. Its impact depends on cultivar, rootstock, climate, virus strain, and infection context, which also complicates symptom-based diagnosis [1,3]. GLD is mainly associated with grapevine leafroll-associated virus 3 (GLRaV-3), a phloem-limited member of the genus *Ampelovirus* that is transmitted through vegetative propagation and by mealybugs and soft scale insects, including the vine mealybug (*Planococcus ficus* Signoret), one of the main vectors in many viticultural regions worldwide [3,4]. Infection is commonly linked with reduced photosynthetic efficiency, poor cane lignification, delayed sugar accumulation, and altered berry composition [3,5]. In Croatia, surveys have shown a high prevalence of GLRaV-3 and other grapevine viruses in autochthonous cultivars, including clones maintained in collections and commercial vineyards, while molecular studies have revealed substantial GLRaV-3 diversity and mixed variant infections within individual vines [6,7,8,9].

Plant viral infections can substantially reprogram host metabolism by altering photosynthesis, carbohydrate partitioning, redox balance, and secondary-metabolite pathways [10,11]. In GLRaV-3-infected vines, transcriptomic and physiological studies have reported down-regulation of photosynthesis-related genes, altered chlorophyll fluorescence, and changes in sugar metabolism, indicating impaired carbon assimilation and transport [2,12,13]. Virus-induced metabolic disturbance is also frequently accompanied by reactive oxygen species (ROS) accumulation and oxidative stress, reflected in changes in hydrogen peroxide (H_2_O_2_), malondialdehyde (MDA), antioxidant systems, and proline metabolism [11,14,15,16].

Phenolic compounds and volatile organic compounds (VOCs) are two major classes of defense-related secondary metabolites in grapevine. Phenolics contribute to antioxidant capacity, cell-wall reinforcement, and antimicrobial or antiviral defense, and grapevine tissues contain diverse phenolic acids, flavonols, flavan-3-ols, stilbenes, and proanthocyanins whose composition depends on genotype and environment [17,18,19]. In grapevine pathosystems, GLRaV-3, grapevine red blotch virus, and xylem-associated pathogens can modify foliar phenolic profiles, supporting the role of both constitutive and inducible phenolics in defense responses [18,20,21,22]. VOCs emitted from leaves and berries provide an additional layer of defense and communication by affecting pathogens, herbivores, vectors, and natural enemies [23,24]. In grapevine, disease pressure and cultivar background can modify VOC profiles, and stress-related compounds such as green leaf volatiles (GLVs) and methyl salicylate (MeSA) are well-established components of direct and systemic defense signaling [23,25,26,27,28,29,30].

Despite this knowledge, integrated leaf-level information on GLRaV-3-associated changes in oxidative stress, phenolic metabolism, and VOC profiles remains limited. Most available studies have focused either on physiological responses, transcriptomic changes, or berry and wine composition, while the combined response of non-volatile and volatile defense-related metabolites in grapevine leaves is still insufficiently characterized [11,13,20]. What is fundamentally new in the present study is the simultaneous assessment of photosynthetic pigments, oxidative stress markers, targeted phenolic compounds, and leaf VOCs in molecularly confirmed GLRaV-3-infected and virus-free plants of the Croatian autochthonous cultivar ‘Plavac mali’. This design allows evaluation of whether GLRaV-3 infection is associated with a coordinated defense-oriented metabolic signature in leaves rather than isolated changes in individual metabolite groups.

Therefore, the aim of this study was to characterize GLRaV-3-associated metabolic responses in grapevine leaves by comparing virus-free and GLRaV-3-infected plants with respect to (i) photosynthetic pigments, (ii) oxidative stress markers (MDA, H_2_O_2_, and proline), (iii) individual phenolic compounds, and (iv) semi-quantitative VOC profiles, including GLVs and MeSA. By integrating targeted chromatographic analyses with multivariate statistics, this work seeks to clarify GLRaV-3-associated changes in leaf metabolism and to provide a metabolite-level description of virus-associated stress and defense responses.

## 2. Materials and Methods

### 2.1. Plant Material, Virus Infection/Detection, and Experimental Design

Self-rooted, virus-free grapevine cuttings of *Vitis vinifera* L. cv. ‘Plavac mali’ were grown under greenhouse conditions. Plant material consisted of cuttings of a single registered clone of *V. vinifera* L. cv. ‘Plavac mali’ [PMC-012], obtained from a registered mother vine plantation used for the production of certified planting material located at Experimental Station Jazbina, Zagreb, Croatia. The use of clonal material from a controlled source minimized genetic variability among biological replicates. Each rooted cutting was grown as an individual plant and represented one independent biological replicate. Grapevine cv. ‘Grk’, previously confirmed by ELISA and RT-qPCR to be infected only with GLRaV-3, was used as the inoculum source. During the first growing season, plants were infected with GLRaV-3 using vine mealybugs (*Planococcus ficus* Signoret) with 5-day acquisition and inoculation access periods. Metabolite sampling for the present study was performed approximately one year after GLRaV-3 inoculation, during the following growing season. After controlled inoculation and confirmation of infection status, virus-free and GLRaV-3-infected plants were maintained together in the same insect-free greenhouse compartment, on the same side of the greenhouse, and on the same bench, using identical pots, substrate, irrigation, and plant management practices. The insect-free conditions prevented secondary vector-mediated GLRaV-3 transmission and allowed both treatment groups to be kept under the same local greenhouse environment. Although a formal randomized block design was not applied, this common greenhouse placement and standardized cultivation minimized treatment-related positional and microclimatic effects. Substrate volumetric water content (VWC%) was monitored as an indicator of pot moisture status and was maintained within 35–40% in both treatment groups. Plants were grown under natural daylight without supplemental artificial lighting, and the natural photoperiod on the sampling date was approximately 15 h 37 min of daylight, calculated for the experimental location. Greenhouse temperature during the 14 days preceding metabolite sampling was summarized using average daily minimum and maximum temperatures; the mean Tmin was 19.4 °C and the mean Tmax was 33.3 °C. At the time of metabolite sampling, plants were in active vegetative growth, corresponding approximately to BBCH 35–39 under greenhouse conditions. To minimize variation related to leaf age, position, and diurnal changes in metabolite levels, sampling was performed for all plants on the same day, 18 June 2025, between 10:00 and 12:00 h. Fully expanded leaves of comparable physiological age were collected from the fifth and sixth nodes from the shoot apex. Before the second growing season, plant infection status was verified by RT-qPCR (Applied Biosystems 7500, Thermo Fisher Scientific, Waltham, MA USA). RNA was extracted from 0.1 g of leaf petioles using the GES method [31], and GLRaV-3 was detected using the FPST assay [32]. Viral status was reconfirmed immediately before metabolite sampling using petiole tissue from the same leaves. At the time of metabolite sampling, GLRaV-3-infected plants did not show visible leafroll symptoms, although the ‘Plavac mali’ clone used in this study is known to develop clearly expressed symptoms later in the growing season. Because visual symptom expression in GLRaV-3-infected grapevines can vary depending on cultivar, phenological stage, environmental conditions, and year, infection status was determined by RT-qPCR rather than by symptom observation. Only RT-qPCR-positive plants were included in the infected group, whereas virus-free controls were RT-qPCR negative.

### 2.2. Determination of Photosynthetic Pigments and Oxidative Stress Markers

Photosynthetic pigments were extracted from leaf tissue with cold 80% (*v*/*v*) aqueous acetone, and all samples were kept on ice throughout the procedure to minimize pigment degradation. After centrifugation to remove debris, absorbance of the clear supernatant was measured at 441, 646, and 663 nm using a UV–Vis spectrophotometer, and concentrations of chlorophyll a, chlorophyll b, and total carotenoids were calculated from these absorbance values using the standard equations of Nayyar and Gupta [33].

Malondialdehyde (MDA), as an indicator of lipid peroxidation, was determined using a thiobarbituric acid (TBA) assay. Leaf tissue was extracted in 0.1% (*w*/*v*) trichloroacetic acid (TCA), an aliquot of the supernatant was mixed with TBA–TCA reagent, the mixture was heated at 95 °C to allow formation of the MDA–TBA adduct, cooled and centrifuged, and absorbance was read at 532 and 600 nm; MDA concentration was then calculated from the corrected absorbance according to the method described by Toth et al. [34].

Hydrogen peroxide content was determined spectrophotometrically according to Velikova et al. [35]. Leaf tissue was extracted in TCA, the supernatant was mixed with potassium phosphate buffer and potassium iodide, incubated, and absorbance of the reaction mixture was recorded at 390 nm to calculate hydrogen peroxide concentration.

Free proline content was quantified using the ninhydrin-based colorimetric method of Bates et al. [36]. Proline was extracted from leaf tissue with 70% ethanol, and the supernatant was reacted with acidic ninhydrin reagent and incubated at 95 °C for color development, then cooled and measured at 520 nm, with proline concentration calculated from a standard curve.

### 2.3. Analysis of Phenolic Compounds in Leaves

Analysis of phenolic compounds in leaves was performed according to Štambuk et al. [22], with minor adaptations for the present experiment. Briefly, 20 mg of powdered sample was mixed with 1.5 mL of extraction solvent composed of acetonitrile–water–formic acid (20:79:1, *v*/*v*/*v*). Extraction was carried out on a Thermomixer C (Eppendorf, Hamburg, Germany) at 60 °C and 600 rpm for 2 h. Following extraction, samples were filtered through a 0.20 μm PTFE syringe filter (Phenex, Phenomenex, Torrance, CA, USA) and subsequently subjected to chromatographic analysis. Polyphenolic compounds were separated, identified, and quantified using an Agilent 1100 Series high-performance liquid chromatography system (Agilent, Waldbronn, Germany), equipped with an autosampler, column thermostat, diode array detector (DAD), and fluorescence detector (FLD), controlled by ChemStation software Rev. B.01.03. Chromatographic separation was performed on a reversed-phase Luna Phenyl-Hexyl column (4.6 × 250 mm, 5 μm particle size) with a corresponding Phenyl guard column (4.0 × 3.0 mm), maintained at 50 °C, following the method described by Tomaz and Maslov [37]. The mobile phases consisted of water–phosphoric acid (99.5:0.5, *v*/*v*; eluent A) and acetonitrile–water–phosphoric acid (50:49.5:0.5, *v*/*v*/*v*; eluent B), delivered at a flow rate of 0.9 mL min^−1^. The gradient program for eluent B was: 0 min, 0%; 7 min, 20%; 35 min, 40%; 40 min, 40%; 45 min, 80%; 50 min, 100%; and 60 min, 0%. The injection volume was 20 μL. Detection was performed using DAD in the range of 200–700 nm, with specific wavelengths applied for different compound classes: flavonol glycosides at 360 nm, hydroxycinnamic acids at 320 nm, stilbenes at 308 nm, and hydroxybenzoic acids at 280 nm. Flavan-3-ols were detected using FLD (λ_ex = 225 nm; λ_em = 320 nm). Compound identification was achieved by comparing retention times and UV–Vis spectra with those of authentic reference standards. Quantification was performed using external calibration curves based on peak areas of corresponding standards. Results are expressed as mg kg^−1^ of dry weight (DW) of grapevine leaves.

The applied HPLC-DAD/FLD method was previously validated for phenolic compound analysis, including calibration linearity, limits of detection (LOD), limits of quantification (LOQ), repeatability, and reproducibility. To improve transparency of phenolic quantification in the present study, calibration linearity expressed as regression coefficient R^2^, LOD, and LOQ values for the standards used for quantification are provided in Supplementary Table S1. Calibration curves were prepared using external standards, and previously reported validation parameters confirmed good linearity and sensitivity of the method.

For each biological replicate, phenolic analysis was performed in three technical replicates. Technical replicate values were first averaged to obtain a single value for each biological replicate, and these biological-replicate means were subsequently used for statistical analysis. Technical replicates were, therefore, used to improve analytical reliability and were not treated as independent biological observations.

### 2.4. Analysis of Volatile Organic Compounds

Analysis of volatile organic compounds (VOCs) in leaves was performed following Štambuk et al. [26], with minor modifications. Briefly, 100 mg of powdered sample was transferred into 20 mL headspace screw-top vials sealed with PTFE/silicone septa and analyzed using an RSH TriPlus autosampler (Thermo Fisher Scientific Inc., Brookfield, WI, USA). Samples were first incubated at 60 °C for 20 min, after which a DVB/CWR/PDMS SPME-Arrow fiber (120 µm × 20 mm; Thermo Fisher Scientific Inc., Brookfield, WI, USA) was exposed to the headspace for 49 min. Subsequently, the fiber was thermally desorbed in the GC injector operating in splitless mode at 250 °C for 10 min. Separation and detection of VOCs were carried out using a TRACE™ 1300 gas chromatograph coupled to an ISQ 7000 TriPlus quadrupole mass spectrometer (Thermo Fisher Scientific Inc., Bartlesville, OK, USA), equipped with a TG-WAXMS A capillary column (60 m × 0.25 mm × 0.25 µm film thickness). Helium was used as the carrier gas at a constant flow rate of 1 mL min^−1^. The oven temperature program started at 40 °C (held for 5 min), followed by a gradual increase of 2 °C min^−1^ up to 210 °C, where it was maintained for an additional 10 min. Mass spectra were acquired in electron impact (EI) mode at 70 eV, scanning a mass range from 30 to 300 m/z. Data acquisition and processing were performed using the Chromeleon™ Data System (Thermo Fisher Scientific Inc., Bartlesville, OK, USA).

Prior to the analytical sequence, the GC–MS system was auto-tuned, the ion source was cleaned, and analyses were performed using a new SPME-Arrow fiber. Standard mixtures and standardized in-house QC samples were included in the analytical sequences to monitor retention-time stability, detector response, extraction repeatability, and overall instrumental performance.

Compound identification was performed by comparing acquired EI mass spectra with those in the Wiley Registry and NIST spectral libraries, by comparison of retention indices, and, where available, by confirmation with authentic reference standards analyzed under the same chromatographic conditions. Linear retention indices (LRIs) were calculated using a homologous series of n-alkanes analyzed under the same chromatographic conditions, according to the Van den Dool and Kratz equation for temperature-programmed gas chromatography. Calculated LRI values were compared with literature values, NIST/Wiley database values, and previously published in-house LRI data obtained on comparable WAX-type columns. The LRI values and identification criteria are provided in Supplementary Table S2.

VOCs were expressed as GC–MS peak areas and interpreted as semi-quantitative relative abundances rather than absolute concentrations. The method was intended for comparative VOC profiling between virus-free and GLRaV-3-infected grapevine leaves analyzed under identical conditions. A liquid internal standard was not used because internal method-development tests in comparable dry grape matrices showed that its addition increased analytical variability, likely due to heterogeneous distribution within the dry matrix and local changes in moisture content, water activity, and analyte partitioning, all of which strongly affect SPME extraction efficiency. Therefore, analytical comparability was ensured by using equal dry sample mass, identical incubation, extraction, and GC–MS conditions, and QC-based monitoring of analytical stability.

For each biological replicate, VOC analysis was performed in three technical replicates. Technical replicate values were first averaged to obtain a single value for each biological replicate, and these biological-replicate means were subsequently used for statistical analysis. Technical replicates were, therefore, used to improve analytical reliability and were not treated as independent biological observations.

### 2.5. Statistical Analysis

Statistical analyses were performed using biological replicates as independent experimental units. Each individual grapevine plant represented one biological replicate. For phenolic compounds and VOCs, three technical replicate measurements were averaged first, and the resulting single mean value per biological replicate was used for statistical analysis. Technical replicates were not treated as independent biological observations.

Virus-free and GLRaV-3-infected leaves were compared using one-way ANOVA. Because only two treatment groups were compared, no post hoc multiple-comparison test was required. Prior to ANOVA, normality of residuals and homogeneity of variances were assessed using the Shapiro–Wilk and Levene’s tests, respectively. No violations of these assumptions were detected for the phenolic and VOC datasets at *p* < 0.05. Exact *p*-values are reported in the corresponding tables.

For phenolic compounds and VOCs, raw ANOVA *p*-values were adjusted for multiple testing using the Benjamini–Hochberg FDR procedure and the Holm–Bonferroni method. FDR-adjusted *p*-values were used as the primary criterion for identifying robust treatment-related metabolite changes, whereas Holm–Bonferroni-adjusted *p*-values were reported as a conservative additional reference. In addition to statistical significance, treatment effects were summarized using fold changes and 95% confidence intervals. Fold change was calculated as the ratio between the mean value in GLRaV-3-infected leaves and the mean value in virus-free leaves. The 95% confidence intervals were calculated for the mean difference between GLRaV-3-infected and virus-free leaves to indicate the precision of treatment-effect estimates.

Principal component analysis (PCA) was performed separately for phenolic and VOC datasets using standardized variables. Prior to PCA, variables were mean-centered and scaled to unit variance, and PCA was performed using the Pearson correlation matrix in XLSTAT. PCA was used as an exploratory unsupervised method to visualize treatment-related variation in metabolite profiles. The PCA results were presented as loading plots/correlation circles rather than sample score plots. These plots visualize the relationships among phenolic compounds or VOCs and indicate which variables contributed most strongly to the principal component structure; therefore, confidence ellipses, which are applicable to sample score plots, were not included. The percentage of variance explained by the first two components was reported in the corresponding figure captions. To complement univariate statistics and unsupervised PCA, exploratory partial least squares-discriminant analysis (PLS-DA) was performed separately for the phenolic and VOC datasets using treatment status as the categorical response variable. Prior to PLS-DA, variables were mean-centered and scaled to unit variance. PLS-DA score plots with 95% confidence ellipses were used to visualize treatment-related separation between virus-free and GLRaV-3-infected leaves. Variable importance in projection (VIP) scores were calculated to identify compounds contributing most strongly to treatment separation. Compounds with VIP scores > 1 were considered the main discriminant variables. Because the study was not designed as a diagnostic-validation study and did not include an independent validation set, PLS-DA/VIP results were interpreted as exploratory support for treatment-associated discriminant metabolites rather than as biomarker validation.

## 3. Results

Virus infections are known to reprogram host plant metabolism in a highly integrated manner, affecting source–sink relationships, carbon allocation, and redox balance at the whole-leaf level [11]. In grapevine, phloem-limited viruses such as GLRaV-3 convert infected leaves into strong metabolic sinks, thereby constraining photosynthetic performance while simultaneously fueling viral replication and defense-related metabolism [2]. Consequently, alterations in photosynthetic pigments and oxidative status are central manifestations of virus-associated physiological imbalance and provide a useful framework for interpreting subsequent changes in phenolic compounds and volatile organic compounds (VOCs). Therefore, changes in photosynthetic pigments and oxidative status are examined first, followed by phenolic and VOC profiles and their integrated interpretation.

### 3.1. Effects of GLRaV-3 on Pigments and Oxidative Status of Leaves

Virus infections frequently disrupt the balance between photosynthetic light capture and downstream metabolic sinks, resulting in changes in pigment composition and increased generation of reactive oxygen species (ROS) in leaves [10,11]. In grapevine, GLRaV-3 has been linked to reduced photosynthetic efficiency and altered carbon allocation, conditions often accompanied by oxidative stress and activation of antioxidant and osmoprotective mechanisms [2,13]. To assess whether these processes occur in our experimental system, photosynthetic pigments (chlorophyll a, chlorophyll b, and total carotenoids) and key oxidative stress markers (MDA, hydrogen peroxide, and free proline) were quantified in virus-free and GLRaV-3-infected grapevine leaves (Table 1). GLRaV-3-infected leaves contained significantly lower amounts of chlorophyll a, chlorophyll b, and total carotenoids, but higher levels of MDA, hydrogen peroxide, and free proline compared with virus-free vines, as confirmed by ANOVA (*p* < 0.05). The reduction in pigment content was moderate but consistent across all chlorophyll fractions, while oxidative stress indicators showed a clear increase, with MDA and proline rising by approximately one third relative to virus-free controls.

**Figure 1:**
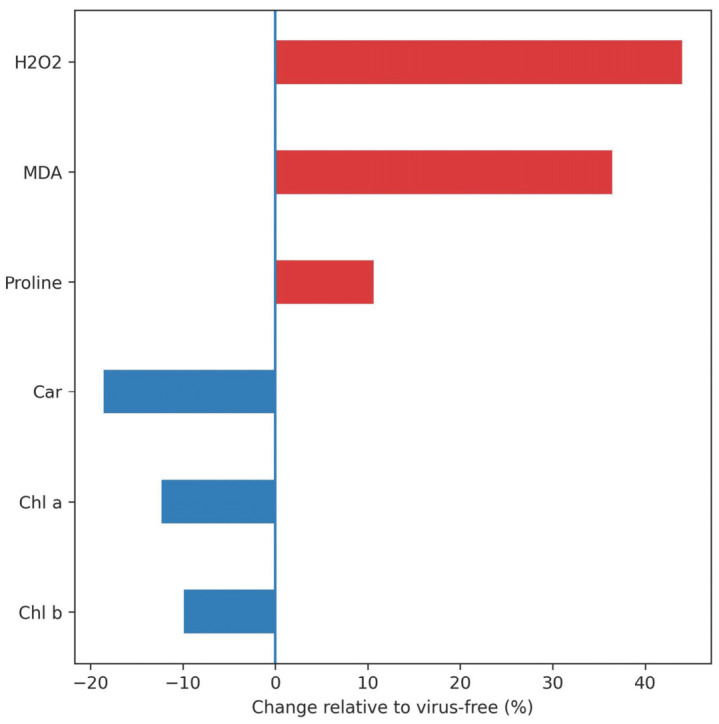
Relative changes (%) in photosynthetic pigments and oxidative stress markers in GLRaV-3-infected grapevine leaves compared with virus-free controls. Blue bars indicate parameters that are decreased in GLRaV-3-infected leaves relative to virus-free controls, whereas red bars indicate parameters that are increased in GLRaV-3-infected leaves. The concurrent increase in H_2_O_2_ and MDA is consistent with enhanced ROS accumulation and lipid peroxidation, as reported for virus-induced oxidative imbalance in grapevine and other plant species [14,15,35]. Hydrogen peroxide may contribute to defense-related signaling but also reflects oxidative injury, while elevated MDA indicates intensified membrane lipid peroxidation and associated physiological costs [11,14,15,16].

To facilitate comparison of the magnitude and direction of virus-induced effects across variables with different units, relative changes are summarized in Figure 1. The reduction in chlorophylls and carotenoids indicates impairment of the photosynthetic apparatus in infected vines, consistent with earlier observations of decreased photosynthetic efficiency, altered chlorophyll fluorescence, and reduced CO_2_ assimilation in GLRaV-3-infected grapevines [2,13]. Because chlorophylls are directly involved in light harvesting and energy transfer, their decline suggests a lower capacity for photochemistry, while reduced carotenoid levels may weaken intrinsic photoprotection and promote ROS formation in chloroplasts [3,5,16,33].

The increase in free proline in infected leaves likely represents an adaptive osmoprotective and antioxidant response, as proline can stabilize proteins and membranes, buffer cellular redox potential, and contribute to ROS detoxification [15,16,36,38]. Overall, the combined decrease in photosynthetic pigments and increase in ROS-related markers indicate that GLRaV-3 infection is associated with a sustained oxidative-stress phenotype, providing a physiological context for the phenolic and VOC changes described below.

### 3.2. Leaf Phenolic Profile and Phenylpropanoid Pathway Remodeling Under GLRaV-3 Infection

**Figure 2:**
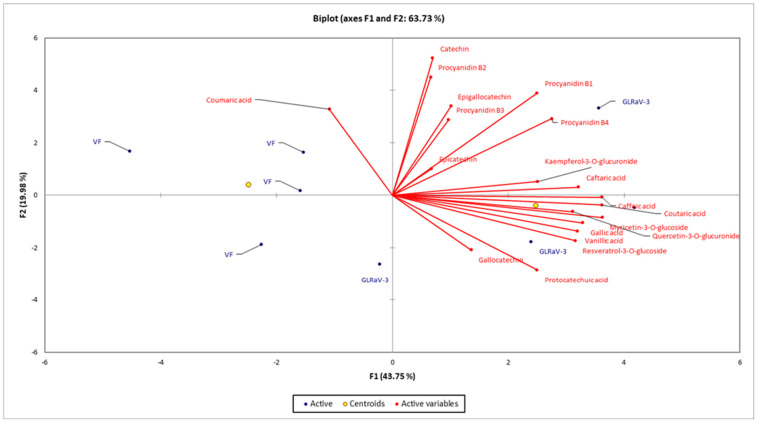
Principal component analysis (PCA) loading plot/correlation circle of leaf phenolic compounds in virus-free (VF) and GLRaV-3-infected grapevine leaves. F1 and F2 together explain 63.73% of the total variance. The plot shows the relationships among phenolic variables and their contributions to the principal component structure.

The GLRaV-3-associated changes in pigment composition and redox balance were accompanied by a clear modification of the leaf phenolic profile. Virus-free (VF) and GLRaV-3-infected leaves differed in several phenolic acids, flavonols, stilbenes, and flavan-3-ols (Table 2), while multivariate analysis summarized treatment-related separation among phenolic variables (Figure 2). Complete statistical output, including FDR-adjusted q-values, Holm–Bonferroni-adjusted *p*-values, effect-size estimates, and 95% confidence intervals, is provided in Supplementary Table S3. The main pattern was an increase in several phenolic acids and flavonols in infected leaves, including myricetin-3-O-glucoside, quercetin-3-O-glucuronide, kaempferol-3-O-glucuronide, caftaric, coutaric, caffeic, gallic, and vanillic acids, resveratrol-3-O-glucoside, and procyanidin B4. In contrast, p-coumaric acid and selected flavan-3-ols showed the opposite or weaker trends, indicating a selective phenolic response rather than a uniform increase in all compound classes [39,40].

The PCA loading plot/correlation circle of phenolic variables (Figure 2) further summarizes the multivariate structure of the phenolic dataset, with the first two principal components explaining 63.73% of the total variability. Most phenolic acids, flavonols, and resveratrol-3-O-glucoside were located on the positive side of F1, indicating that this component primarily reflects variables more strongly associated with GLRaV-3-infected leaves. In contrast, several flavan-3-ols and procyanidins were positioned closer to F2 or nearer the origin, while p-coumaric acid was separated toward negative F1, consistent with its higher abundance in virus-free leaves. Thus, PCA supports the univariate results by showing that selected flavonols, hydroxycinnamic and hydroxybenzoic acids, and stilbene-related compounds were the main contributors to the GLRaV-3-associated phenolic profile. Similar multivariate discrimination based on foliar phenolics has been reported in other grapevine pathosystems, including phytoplasma infection and *Xylella fastidiosa* [18,41].

Exploratory PLS-DA further supported treatment-related separation of the phenolic dataset, with 95% confidence ellipses indicating distinct clustering of virus-free and GLRaV-3-infected leaves (Supplementary Figure S1). VIP analysis identified kaempferol-3-O-glucuronide, myricetin-3-O-glucoside, gallic acid, quercetin-3-O-glucuronide, coutaric acid, procyanidin B4, epicatechin, caffeic acid, caftaric acid, vanillic acid, resveratrol-3-O-glucoside, and procyanidin B1 as the main discriminant phenolic variables (VIP > 1; Supplementary Figure S2).

Together, the univariate, PCA, and PLS-DA results indicate that GLRaV-3 infection is associated with a shift in phenolic composition toward compounds with recognized antioxidant, structural, and antimicrobial roles in grapevine. Flavonols such as quercetin, myricetin, and kaempferol derivatives are effective ROS scavengers and contribute to UV screening, while phenolic acids and stilbenes, including resveratrol-3-O-glucoside, are associated with cell-wall reinforcement and antimicrobial defense [22,39,40,42,43]. However, because enzyme activities and gene expression were not measured, these data should be interpreted as evidence of altered metabolite accumulation rather than direct proof of pathway activation or metabolic flux. The lower level of p-coumaric acid may indicate altered pathway allocation toward downstream derivatives, but this remains inferential and should be tested using transcriptomic, enzymatic, or isotope-labeling approaches [14,41,44].

### 3.3. Leaf Volatile Organic Compounds Under GLRaV-3 Infection

Volatile organic compound (VOC) profiles of virus-free and GLRaV-3-infected leaves showed pronounced treatment-related differences (Table 3). The VOC dataset is presented as semi-quantitative GC-MS peak-area-based relative abundance, and the complete statistical output, including FDR-adjusted q-values, Holm–Bonferroni-adjusted *p*-values, effect size estimates, and 95% confidence intervals, is provided in Supplementary Table S4. GLRaV-3-infected leaves showed higher relative abundance of several fatty acid-derived aldehydes, alcohols, and esters, including 2-hexenoic acid, 2-pentenal, 2-heptenal, 2-octenal, 2-nonenal, 2,4-heptadienal, 2,4-hexadienal, 2,6-nonadienal, hexanal, nonanal, octanal, 1-penten-3-ol, 3-hexen-1-ol, and 3-hexen-1-yl acetate. Additional compounds, including 3-methoxy-1-butanol, 1-hexanol, 1-methoxy-2-propanol, hexyl acetate, benzaldehyde, decanal, dihydromyrcenol, and methyl salicylate, were also higher in infected leaves, whereas 1-pentanol, benzyl alcohol, cyclocitral, geranylacetone, phenylacetaldehyde, and propanoic acid were more abundant in virus-free leaves. This pattern indicates selective reorganization of leaf VOC profiles rather than a general increase in all volatile classes.

**Figure 3:**
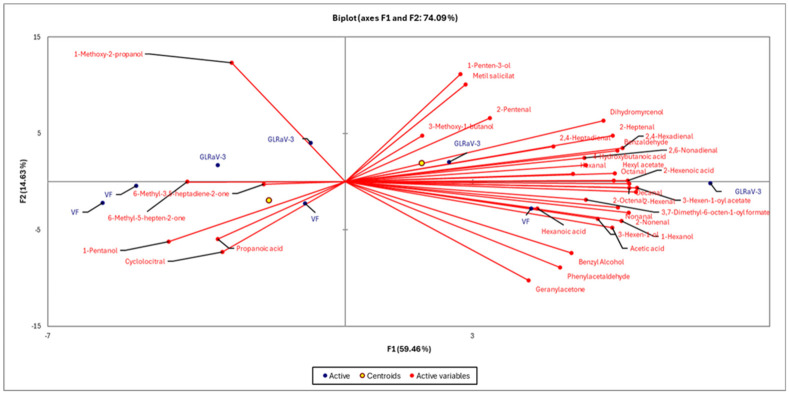
Principal component analysis (PCA) loading plot/correlation circle of leaf VOCs in virus-free (VF) and GLRaV-3-infected grapevine leaves. F1 and F2 explain 59.46% and 14.63% of total variance, respectively, together accounting for 74.09%. The plot visualizes relationships among VOC variables and their contribution to the principal component structure.

The multivariate structure of the VOC dataset is shown by the PCA loading plot (correlation circle) in Figure 3, where the first principal component (F1, 59.46% of variance) and the second component (F2, 14.63%) together explain 74.09% of the total variability in VOC profiles. Most VOCs that were higher in GLRaV-3-infected leaves, particularly C6 and C9 aldehydes, alcohols, and esters such as 2-hexenal, hexanal, 2-pentenal, 2-heptenal, 2-octenal, 2-nonenal, 2,4-heptadienal, 2,4-hexadienal, 2,6-nonadienal, 3-hexen-1-ol, 3-hexen-1-yl acetate, hexyl acetate, benzaldehyde, dihydromyrcenol, nonanal, and decanal, were positioned on the positive side of F1. This indicates that F1 largely represents the group of infection-associated VOC variables. In contrast, VOCs that were relatively more abundant in VF leaves, including 1-pentanol, cyclocitral, and propanoic acid, were positioned toward negative F1. The F2 axis further separated compounds such as 1-methoxy-2-propanol, 1-penten-3-ol, and methyl salicylate, which contributed strongly to the GLRaV-3-related VOC signature. Overall, PCA complements the univariate results by showing that GLRaV-3 infection is associated with a coordinated VOC profile characterized mainly by lipid-derived GLVs and selected benzenoid/salicylate-related compounds.

Exploratory PLS-DA further supported treatment-related separation of the VOC dataset, with 95% confidence ellipses indicating distinct clustering of virus-free and GLRaV-3-infected leaves (Supplementary Figure S3). VIP analysis identified 1-methoxy-2-propanol, dihydromyrcenol, 1-pentanol, 1-penten-3-ol, 2,4-hexadienal, methyl salicylate, cyclocitral, hexanal, 2-hexenoic acid, 4-hydroxybutanoic acid, geranylacetone, propanoic acid, 2-pentenal, 2,4-heptadienal, and 2-heptenal as the VOC variables contributing most strongly to treatment separation (VIP > 1; Supplementary Figure S4).

In the context of the current grapevine VOC literature, this GLRaV-3-associated pattern supports the view that defense-related VOCs form stimulus-specific blends rather than uniform stress responses. Grapevine responses to pathogens, beneficial microorganisms, and elicitors often involve GLVs, benzenoids, and terpenoids in combinations that depend on the eliciting agent, plant organ, and environmental conditions [45,46]. The higher relative abundance of C6 aldehydes and alcohols, together with methyl salicylate, is, therefore, consistent with a VOC profile associated with defense-related signaling, although direct hormonal measurements were not performed [46,47,48,49].

The higher relative abundance of GLVs and methyl salicylate in infected leaves may indicate overlapping defense-related processes. GLVs such as 2-hexenal and hexanal can inhibit fungal growth, influence herbivore behavior, and prime neighboring tissues or plants for enhanced defense responses [45,47,50]. Methyl salicylate is associated with salicylic acid-related signaling and systemic acquired resistance, and its increase here supports a metabolite-level link between GLRaV-3 infection and salicylate-related metabolism that should be tested by phytohormone and gene-expression analyses [46,49].

The concurrent decrease in benzyl alcohol, phenylacetaldehyde, geranylacetone, and cyclocitral indicates altered allocation within shikimate- and carotenoid-derived VOC metabolism. Benzenoids share upstream precursors with salicylic acid and other defense-related metabolites, whereas carotenoid-derived apocarotenoids are linked to signaling, photoprotection, and aroma. Their lower abundance in infected leaves may, therefore, reflect changes in chorismate- and carotenoid-derived metabolism, consistent with shifts reported after elicitor treatment or pathogen attack in grapevine [25,45,47,49].

These GLRaV-3-associated VOC changes complement the non-volatile phenolic responses observed in the same plants and support the view that infection alters multiple branches of secondary metabolism. However, the data remain correlative and do not demonstrate the underlying regulatory mechanisms; combined phenolic and VOC profiles should, therefore, be interpreted as metabolite-level indicators of infection-associated reprogramming rather than direct evidence of enzymatic or hormonal pathway activation [2,49].

From an applied perspective, the GLRaV-3-associated VOC pattern documented in Table 3 and Figure 3 highlights treatment-associated metabolic features that may guide future targeted studies. Because no independent validation set or diagnostic classification framework was used, these VOCs should not be interpreted as validated biomarkers. Their broader relevance requires validation across cultivars, seasons, infection stages, and field conditions.

## 4. Discussion

GLRaV-3-associated changes in pigments, oxidative status, phenolics, and VOCs indicate a coherent, multi-layered adjustment of grapevine leaf metabolism. At the level of primary physiology, infected leaves showed reduced chlorophyll a, chlorophyll b, and carotenoids, consistent with impaired light harvesting and altered photosynthetic performance previously reported for GLRaV-3-infected grapevines [2,13]. This pigment loss coincided with elevated H_2_O_2_, MDA, and proline, indicating oxidative imbalance and osmoprotective adjustment. Similar virus-induced changes in ROS-related metabolism have been described in other plant-virus systems, where reduced photosynthetic capacity and altered carbon allocation can contribute to oxidative stress and the accumulation of defense-related metabolites [10,11,51].

This altered physiological background provides a plausible context for the phenolic profile observed in infected leaves. GLRaV-3-infected leaves accumulated higher levels of several hydroxycinnamic and hydroxybenzoic acids, quercetin-, myricetin-, and kaempferol-based flavonols and resveratrol-3-O-glucoside, suggesting changes in phenylpropanoid-related metabolite pools. Because PAL, C4H, 4CL, CHS, F3H, FLS, and STS activities or transcript levels were not measured, these results should not be interpreted as direct evidence of enzyme activation [11,39].

A similar interpretation applies to volatile metabolism. The higher relative abundance of GLV-related compounds and methyl salicylate, together with lower levels of selected benzenoids and apocarotenoids, is consistent with altered lipid-, salicylate-, chorismate- and carotenoid-derived VOC profiles. However, direct flux measurements were not conducted; therefore, these patterns indicate altered metabolite allocation rather than proof of metabolic rerouting [26,52].

Viewed more broadly, reduced pigment content, elevated oxidative stress markers, increased phenolic compounds, and a VOC profile enriched in GLVs and methyl salicylate suggest that GLRaV-3 infection shifts leaves toward an infection-associated chemical profile. Such changes may impose physiological costs while increasing the abundance of compounds with antioxidant, antimicrobial, or signaling functions, consistent with the complex role of viral infection in shaping plant responses to additional stresses [2,10,11,52].

The overlap between GLRaV-3-associated metabolites and compounds involved in other grapevine defense responses suggests that chronic viral infection may influence subsequent plant-pathogen interactions. However, this remains a hypothesis that should be tested under controlled co-infection or pathogen-challenge conditions, particularly because the present study did not measure downstream disease outcomes.

**Figure 4:**
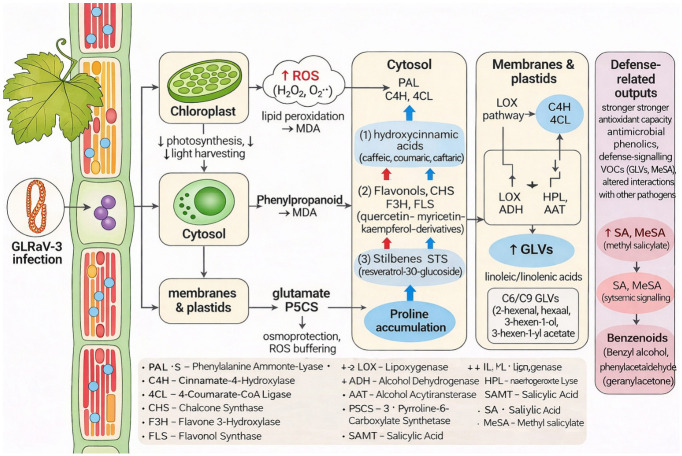
Schematic model of proposed GLRaV-3-associated metabolic changes in grapevine leaves, linking reduced photosynthetic pigments and increased ROS formation with changes in phenylpropanoid-derived metabolites and volatile organic compound profiles. The model is based on metabolite-level observations and should be interpreted as a working hypothesis rather than direct evidence of pathway activation.

The compounds that most consistently distinguished GLRaV-3-infected leaves from virus-free leaves, including selected flavonols, stilbenes, GLVs, and methyl salicylate, should be considered GLRaV-3-associated metabolic features requiring further validation. Validation across cultivars, seasons, symptom stages, virus titers, and field environments will be necessary before these metabolites can be used as reliable indicators of GLRaV-3 status or as selection targets. The proposed relationships among pigment loss, oxidative stress, phenolic accumulation, and VOC changes are summarized in Figure 4.

A limitation of this study is that it was conducted on a single autochthonous cultivar, ‘Plavac mali’, under greenhouse conditions with molecularly confirmed but, at the moment of sampling, visually asymptomatic GLRaV-3 infection. In addition, although greenhouse conditions, pot moisture, irrigation, plant management, and plant placement were standardized across treatments, the lack of a formal randomized block design means that subtle positional or microclimatic effects within the greenhouse cannot be entirely excluded. Although no visible symptoms were observed at the time of sampling, the ‘Plavac mali’ clone examined in this study has consistently exhibited pronounced GLRaV-3 symptoms later in the growing season, indicating that the analyzed plants represented a pre-symptomatic stage of infection rather than a symptomless interaction. The absence of visible symptoms at sampling should, therefore, be considered when interpreting the results, as GLRaV-3 symptom expression may vary depending on phenological stage, environmental conditions, and year, even in chronically infected vines. As a result, the magnitude and direction of the observed metabolite changes may differ in other cultivars, under field conditions, in symptomatic leaves, or under different environmental regimes. Viral load was not used as a continuous explanatory variable because Ct values were recorded for diagnostic confirmation but were not directly paired with each analytical aliquot used for phenolic and VOC profiling. Future studies should combine paired viral load quantification, symptom scoring, transcriptomics, enzyme assays, and time-resolved metabolomics across multiple cultivars and seasons to determine which responses are general features of GLRaV-3 infection and which are cultivar- or environment-dependent.

## 5. Conclusions

GLRaV-3 infection in grapevine leaves was associated with coordinated physiological and metabolite-level changes, including lower chlorophyll a, chlorophyll b, and carotenoid contents; higher hydrogen peroxide, malondialdehyde, and proline levels; and distinct shifts in phenolic and volatile organic compound profiles. Because photosynthetic performance was not directly measured, the observed decrease in photosynthetic pigments suggests a likely reduction in photosynthetic capacity rather than demonstrating it directly. The accumulation of selected hydroxycinnamic and hydroxybenzoic acids, flavonols, and stilbenes, along with changes in green leaf volatiles and methyl salicylate, is consistent with altered phenylpropanoid- and lipid-derived volatile metabolism and a more defense-associated leaf chemical phenotype. However, enzyme activities, gene expression, and metabolic fluxes were not measured; therefore, these pathway-level interpretations should be regarded as hypotheses generated from metabolite patterns rather than direct mechanistic evidence. By integrating pigment and oxidative stress markers with targeted phenolic and VOC profiling, this study offers a comprehensive metabolite-level view of GLRaV-3-associated changes in grapevine leaves. The metabolites and volatile signatures identified here should, therefore, be interpreted as GLRaV-3-associated metabolic features rather than validated biomarkers, and their broader relevance requires validation across cultivars, infection stages, symptom expression levels, and environmental conditions. Future work should combine paired viral load quantification, time-resolved metabolomics, transcriptomics or proteomics, and enzyme activity measurements to connect metabolite changes more directly with pathway regulation. These findings contribute to a deeper understanding of grapevine–virus interactions and provide a biochemical framework for metabolite-informed diagnostic and resistance-oriented breeding approaches. These findings contribute to a deeper understanding of grapevine–virus interactions and provide a biochemical framework for future metabolite-informed studies of grapevine responses to viral infection.

## Figures and Tables

**Table 1:** Photosynthetic pigment contents and oxidative stress markers (MDA, hydrogen peroxide, and free proline) in leaves of virus-free and GLRaV-3-infected grapevines.

Parameter	Virus-Free	GLRaV-3	ANOVA *p*-Value
Chlorophyll a (mg 100 g^−1^ DW)	657.85 ± 19.74	576.75 ± 17.30	0.0059
Chlorophyll b (mg 100 g^−1^ DW)	314.05 ± 9.42	282.96 ± 8.49	0.0132
Total carotenoids (mg 100 g^−1^ DW)	154.65 ± 4.64	125.89 ± 4.08	0.0062
MDA (nmol g^−1^ DW)	8.73 ± 0.26	11.91 ± 0.36	0.0002
Proline (µmol g^−1^ DW)	7.98 ± 0.24	8.83 ± 0.26	0.0146
Hydrogen peroxide (µmol g^−1^ DW)	0.25 ± 0.01	0.36 ± 0.01	0.0002

**Table 2:** Leaf phenolic profile of virus-free and GLRaV-3-infected grapevine leaves. Values are expressed as mean ± SD (mg kg^−1^ DW). Raw *p*-values were obtained by one-way ANOVA. Fold change is included as an effect-size measure.

Compound	Virus-Free Mean ± SD	GLRaV-3 Mean ± SD	Fold Change GLRaV-3/VF	ANOVA *p*-Value
Myricetin-3-O-glucoside	29.62 ± 0.89	51.90 ± 1.56	1.75	2.75 × 10^−5^
Quercetin-3-O-glucuronide	3217.65 ± 96.53	4771.32 ± 143.14	1.48	9.89 × 10^−5^
Kaempferol-3-O-glucuronide	4.14 ± 0.12	11.64 ± 0.35	2.81	3.96 × 10^−6^
Caftaric acid	5124.70 ± 153.74	5742.48 ± 172.27	1.12	0.0098
Coutaric acid	134.28 ± 4.03	163.49 ± 4.90	1.22	0.0013
Caffeic acid	511.14 ± 15.33	662.46 ± 19.87	1.30	4.75 × 10^−4^
Coumaric acid	105.22 ± 3.16	97.97 ± 2.94	0.93	0.0436
Gallic acid	7.96 ± 0.24	11.19 ± 0.34	1.41	1.70 × 10^−4^
Protocatechuic acid	303.80 ± 9.11	328.80 ± 9.86	1.08	0.0321
Vanillic acid	379.54 ± 11.39	430.88 ± 12.93	1.14	0.0067
Resveratrol-3-O-glucoside	129.96 ± 3.90	148.00 ± 4.44	1.14	0.0061
Gallocatechin	62.84 ± 1.89	68.30 ± 2.05	1.09	0.0274
Epigallocatechin	1411.05 ± 42.33	1419.87 ± 42.60	1.01	0.8117
Procyanidin B1	46.32 ± 1.39	50.60 ± 1.52	1.09	0.0228
Procyanidin B2	30.84 ± 0.93	32.27 ± 0.97	1.05	0.1376
Catechin	27.75 ± 0.83	25.34 ± 0.76	0.91	0.0209
Procyanidin B3	21.55 ± 0.65	22.88 ± 0.69	1.06	0.0714
Procyanidin B4	21.74 ± 0.65	25.01 ± 0.75	1.15	0.0047
Epicatechin	12.90 ± 0.39	11.67 ± 0.35	0.90	0.0149

**Table 3:** Volatile organic compounds in virus-free and GLRaV-3-infected grapevine leaves. Values are expressed as mean ± SD of GC-MS peak area ×10^3^ and represent semi-quantitative relative VOC abundance. Raw *p*-values were obtained by one-way ANOVA. Fold change is included as an effect-size measure.

Compound	Virus-Free Mean ± SD	GLRaV-3 Mean ± SD	Fold Change GLRaV-3/VF	ANOVA *p*-Value
1-Methoxy-2-propanol	3141.16 ± 94.23	5796.30 ± 173.89	1.85	2.03 × 10^−5^
Dihydromyrcenol	99.62 ± 2.99	164.56 ± 4.94	1.65	4.08 × 10^−5^
1-Penten-3-ol	1604.59 ± 48.14	2537.92 ± 76.14	1.58	5.67 × 10^−5^
2,4-Hexadienal	1469.75 ± 44.09	2099.20 ± 62.98	1.43	1.44 × 10^−4^
Hexanal	4614.22 ± 138.43	6569.77 ± 197.09	1.42	1.48 × 10^−4^
2-Pentenal	1098.17 ± 32.95	1531.30 ± 45.94	1.39	1.86 × 10^−4^
Geranylacetone	151.20 ± 4.54	109.05 ± 3.27	0.72	1.99 × 10^−4^
Methyl salicylate	257.63 ± 7.73	354.09 ± 10.62	1.37	2.20 × 10^−4^
1-Pentanol	233.76 ± 7.01	174.86 ± 5.25	0.75	3.10 × 10^−4^
2,4-Heptadienal	1739.67 ± 52.19	2291.47 ± 68.74	1.32	3.78 × 10^−4^
2-Heptenal	196.24 ± 5.89	257.22 ± 7.72	1.31	4.05 × 10^−4^
Propanoic acid	253.78 ± 7.61	194.94 ± 5.85	0.77	4.46 × 10^−4^
4-Hydroxybutanoic acid	33.20 ± 1.00	43.09 ± 1.29	1.30	4.67 × 10^−4^
3-Hexen-1-yl acetate	15,172.31 ± 455.17	19,393.46 ± 581.80	1.28	5.85 × 10^−4^
2-Hexenoic acid	929.15 ± 27.87	1179.13 ± 35.37	1.27	6.54 × 10^−4^
Hexyl acetate	189.40 ± 5.68	239.27 ± 7.18	1.26	7.03 × 10^−4^
6-Methyl-5-hepten-2-one	3136.62 ± 94.10	2509.19 ± 75.28	0.80	8.37 × 10^−4^
Cyclocitral	387.67 ± 11.63	314.19 ± 9.43	0.81	0.0010
2,6-Nonadienal	144.52 ± 4.34	175.34 ± 5.26	1.21	0.0014
Benzaldehyde	602.98 ± 18.09	729.43 ± 21.88	1.21	0.0015
2-Octenal	6330.78 ± 189.92	7640.61 ± 229.22	1.21	0.0016
Octanal	616.58 ± 18.50	729.19 ± 21.88	1.18	0.0024
Phenylacetaldehyde	183.29 ± 5.50	155.06 ± 4.65	0.85	0.0025
2-Nonenal	89.48 ± 2.68	105.08 ± 3.15	1.17	0.0028
2-Hexenal	234,711.18 ± 7041.34	269,233.53 ± 8077.01	1.15	0.0051
Decanal	710.00 ± 21.30	804.17 ± 24.13	1.13	0.0071
3-Hexen-1-ol	4917.99 ± 147.54	5558.85 ± 166.77	1.13	0.0076
3-Methoxy-1-butanol	2130.89 ± 63.93	2387.60 ± 71.63	1.12	0.0098
1-Hexanol	240.09 ± 7.20	268.09 ± 8.04	1.12	0.0109
Benzyl Alcohol	402.74 ± 12.08	364.42 ± 10.93	0.90	0.0152
6-Methyl-3,5-heptadiene-2-one	94.53 ± 2.84	85.78 ± 2.57	0.91	0.0167
Nonanal	2319.96 ± 69.60	2505.85 ± 75.18	1.08	0.0348
Hexanoic acid	165.21 ± 4.96	176.15 ± 5.28	1.07	0.0591
3,7-Dimethyl-6-octen-1-oyl formate	1029.85 ± 30.90	1057.82 ± 31.73	1.03	0.3354
Acetic acid	21.41 ± 0.64	21.85 ± 0.66	1.02	0.4499

## Data Availability

The data presented in this study are available on request from the corresponding author due to the large size of the dataset, which makes it impractical for public distribution.

## Supplementary Materials

The following supporting information can be downloaded at: https://www.mdpi.com/article/10.3390/metabo16060359/s1. Table S1: Calibration linearity and sensitivity parameters of the HPLC-DAD/FLD method for phenolic compound quantification, including regression coefficients (R^2^), limits of detection (LOD), and limits of quantification (LOQ). Table S2: Retention indices and identification parameters for volatile organic compounds detected in grapevine leaves. Table S3: Full statistical output for phenolic compounds, including mean difference, 95% confidence intervals, fold change, ANOVA *p*-values, FDR-adjusted q-values, and Holm–Bonferroni-adjusted *p*-values. Table S4: Full statistical output for VOCs, including mean difference, 95% confidence intervals, fold change, ANOVA *p*-values, FDR-adjusted q-values, and Holm–Bonferroni-adjusted *p*-values. Figure S1. Exploratory PLS-DA score plot of phenolic compounds in virus-free and GLRaV-3-infected grapevine leaves. Variables were mean-centered and scaled to unit variance prior to analysis. Ellipses represent 95% confidence regions for each treatment group. Figure S2. Variable importance in projection (VIP) scores from exploratory PLS-DA of phenolic compounds. Compounds with VIP scores > 1 were considered the main variables contributing to treatment discrimination. Figure S3. Exploratory PLS-DA score plot of volatile organic compounds in virus-free and GLRaV-3-infected grapevine leaves. Variables were mean-centered and scaled to unit variance prior to analysis. Ellipses represent 95% confidence regions for each treatment group. Figure S4. Variable importance in projection (VIP) scores from exploratory PLS-DA of volatile organic compounds. Compounds with VIP scores > 1 were considered the main variables contributing to treatment discrimination.
