# Supplementary material for: Metabolic Responses of Grapevine Leaves to Grapevine Leafroll-Associated Virus 3 Infection
Source: Metabolites. 2026 May 27;16(6):359. doi: 10.3390/metabo16060359 (PMC13303152; doi:10.3390/metabo16060359)
Supplement: Supplementary file 1 [file metabolites-16-00359-s001.zip › Supplemental Table S1.pdf]

Supplemental Table S1. Calibration linearity and sensitivity parameters of the HPLC-DAD/FLD method for phenolic compound quantification. Regression coefficients (R^2), limits of detection (LOD) and limits of quantification (LOQ) are provided for the phenolic standards used for quantification. LOD and LOQ are expressed as mg L^{-1}.

**Compound**	**R^2**	**LOD mg/L**	**LOQ mg/L**
Myricetin-3-O-glucoside	0.9997	0.26	0.79
Quercetin-3-O-glucuronide	0.9999	0.06	0.19
Kaempferol-3-O-glucuronide	0.9998	0.05	0.15
Caftaric acid	0.9978	3.07×10^{-5}	9.21×10^{-5}
Coutaric acid	0.9990	0.08	0.25
Caffeic acid	0.9990	3.01×10^{-5}	9.03×10^{-5}
Coumaric acid	0.9990	0.08	0.25
Gallic acid	0.9999	0.05	0.14
Protocatechuic acid	0.9998	0.05	0.12
Vanillic acid	0.9998	0.04	0.13
Resveratrol-3-O-glucoside	1.0000	0.02	0.06
Gallocatechin	0.9993	3.05×10^{-5}	9.15×10^{-5}
Epigallocatechin	0.9993	3.05×10^{-5}	9.15×10^{-5}
Procyanidin B1	0.9995	0.14	0.42
Procyanidin B2	1.0000	0.14	0.42
Catechin	1.0000	2.96×10^{-5}	8.89×10^{-5}
Procyanidin B3	0.9992	0.13	0.41
Procyanidin B4	0.9997	0.14	0.42
