# Supplementary material for: Metabolic Responses of Grapevine Leaves to Grapevine Leafroll-Associated Virus 3 Infection
Source: Metabolites. 2026 May 27;16(6):359. doi: 10.3390/metabo16060359 (PMC13303152; doi:10.3390/metabo16060359)
Supplement: Supplementary file 1 [file metabolites-16-00359-s001.zip › Supplemental Table S2.pdf]

Supplementary Table S2. Retention indices and identification parameters for volatile organic compounds detected in grapevine leaves. Linear retention indices (LRI) were calculated using a homologous n-alkane series analyzed under the same chromatographic conditions and compared with literature, NIST/Wiley database and previously published in-house LRI values. Compound identification was based on EI mass spectral matching, LRI comparison and authentic standards where available.

Compound name	LRI (det)	LRI (lit)
(E)-2-Hexenoic acid	1935	1941
(E)-2-Pentenal	1079	1073
3-Methoxy-1-butanol	1566	1568
1-Hexanol	1359	1355
1-Pentanol	1413	1410
1-Penten-3-ol	1158	1151
(E,E)-2,4-Heptadienal	1501	1508
2,4-Hexadienal	1434	1434
(E,Z)-2,6-Nonadienal	1590	1595
(E)-2-Heptenal	1310	1314
2-Hexenal	1321	1329
(E)-2-Nonenal	1528	1535
(E)-2-Octenal	1445	1437
1-Methoxy-2-propanol	1167	1160
trans-3-Hexen-1-ol	1724	1725
3-Hexen-1-yl acetate	1333	1327
4-Hydroxybutanoic acid	1640	1646
6-Methyl-5-hepten-2-one	1345	1337
6-Methyl-3,5-heptadien-2-one	1580	1582
3,7-Dimethyl-6-octen-1-yl formate	1630	1629
Acetic acid	1459	1455
Hexyl acetate	1291	1287
Benzaldehyde	1511	1515
Benzyl alcohol	1861	1684
β-Cyclocitral	1601	1598
Decanal	1500	1496
Dihydromyrcenol	1463	1450
Geranylacetone	1841	1838
Hexanal	1075	1075
Hexanoic acid	1849	1843
Nonanal	1399	1395
Octanal	1296	1290
Phenylacetaldehyde	1643	1640
Propanoic acid	1500	1510
Methyl salicylate	1746	1750
