# Supplementary material for: Metabolic Responses of Grapevine Leaves to Grapevine Leafroll-Associated Virus 3 Infection
Source: Metabolites. 2026 May 27;16(6):359. doi: 10.3390/metabo16060359 (PMC13303152; doi:10.3390/metabo16060359)
Supplement: Supplementary file 1 [file metabolites-16-00359-s001.zip › Supplemental Table S3.pdf]

Supplemental Table S3. Leaf phenolic profile of virus-free and GLRaV-3-infected grapevine leaves. Adjusted p-values were calculated using the Benjamini–Hochberg false discovery rate (FDR) and Holm–Bonferroni correction. 95% confidence intervals (CI) are included as effect-size and precision estimates. Bold rows indicate compounds that remained significant after Holm–Bonferroni correction ($p < 0.05$).

**Compound**	**Difference GLRaV-3–VF**	**95% CI for difference**	**FDR q-value**	**Holm p-value**
**Myricetin-3-O-glucoside**	**22.28**	**19.09 to 25.47**	**2.62e-04**	**4.96e-04**
**Quercetin-3-O-glucuronide**	**1553.67**	**1260.90 to 1846.45**	**6.26e-04**	**0.0017**
**Kaempferol-3-O-glucuronide**	**7.50**	**6.73 to 8.26**	**7.52e-05**	**7.52e-05**
Caftaric acid	617.78	245.77 to 989.79	0.0186	0.0978
**Coutaric acid**	**29.20**	**18.88 to 39.53**	**0.0043**	**0.0188**
**Caffeic acid**	**151.31**	**110.03 to 192.59**	**0.0018**	**0.0071**
Coumaric acid	-7.25	-14.18 to -0.32	0.0517	0.1743
**Gallic acid**	**3.23**	**2.54 to 3.92**	**8.06e-04**	**0.0027**
Protocatechuic acid	25.00	3.42 to 46.59	0.0407	0.1674
Vanillic acid	51.34	23.55 to 79.12	0.0141	0.0737
Resveratrol-3-O-glucoside	18.03	8.50 to 27.57	0.0141	0.0737
Gallocatechin	5.46	0.98 to 9.93	0.0372	0.1674
Epigallocatechin	8.82	-87.45 to 105.09	0.8117	0.8117
Procyanidin B1	4.28	0.97 to 7.58	0.0333	0.1674
Procyanidin B2	1.43	-0.72 to 3.58	0.1452	0.2751
Catechin	-2.40	-4.22 to -0.59	0.0331	0.1674
Procyanidin B3	1.33	-0.19 to 2.84	0.0798	0.2141
Procyanidin B4	3.27	1.66 to 4.88	0.0127	0.0609
Epicatechin	-1.23	-2.07 to -0.40	0.0257	0.1338

Note: FDR, false discovery rate; Holm p-value; CI, confidence interval. Difference and 95% CI are calculated as GLRaV-3 minus virus-free. Technical replicates were averaged first, and biological-replicate means were used for statistical analysis.
