# Supplementary material for: Metabolic Responses of Grapevine Leaves to Grapevine Leafroll-Associated Virus 3 Infection
Source: Metabolites. 2026 May 27;16(6):359. doi: 10.3390/metabo16060359 (PMC13303152; doi:10.3390/metabo16060359)
Supplement: Supplementary file 1 [file metabolites-16-00359-s001.zip › Supplemental Table S4.pdf]

Supplemental Table S4. Volatile organic compounds in virus-free and GLRaV-3-infected grapevine leaves. Adjusted p-values were calculated using the Benjamini–Hochberg false discovery rate (FDR) and Holm–Bonferroni procedures. 95% confidence interval (CI) values are expressed as GLRaV-3–VF peak area ×10^3.

Compound	Difference GLRaV-3–VF	95% CI for difference	FDR q-value	Holm p-value
**1-Methoxy-2-propanol**	**2655.14**	**2297.12 to 3013.17**	**6.61e-4**	**7.10e-4**
**Dihydromyrcenol**	**64.94**	**54.85 to 75.03**	**6.61e-4**	**0.0014**
**1-Penten-3-ol**	**933.33**	**777.84 to 1088.83**	**6.61e-4**	**0.0019**
**2,4-Hexadienal**	**629.45**	**500.32 to 758.59**	**9.63e-4**	**0.0046**
**Hexanal**	**1955.55**	**1551.30 to 2359.81**	**9.63e-4**	**0.0046**
**2-Pentenal**	**433.13**	**338.71 to 527.54**	**9.63e-4**	**0.0056**
**Geranylacetone**	**-42.15**	**-51.48 to -32.82**	**9.63e-4**	**0.0058**
**Methyl salicylate**	**96.46**	**74.59 to 118.33**	**9.63e-4**	**0.0062**
**1-Pentanol**	**-58.90**	**-73.40 to -44.41**	**0.0012**	**0.0084**
**2,4-Heptadienal**	**551.80**	**409.41 to 694.18**	**0.0013**	**0.0098**
**2-Heptenal**	**60.98**	**44.98 to 76.98**	**0.0013**	**0.0101**
**Propanoic acid**	**-58.85**	**-74.65 to -43.04**	**0.0013**	**0.0107**
**4-Hydroxybutanoic acid**	**9.88**	**7.20 to 12.57**	**0.0013**	**0.0107**
**3-Hexen-1-oyl acetate**	**4221.15**	**3009.49 to 5432.81**	**0.0015**	**0.0129**
**2-Hexenoic acid**	**249.99**	**176.21 to 323.76**	**0.0015**	**0.0137**
**Hexyl acetate**	**49.88**	**34.89 to 64.86**	**0.0015**	**0.0141**
**6-Methyl-5-hepten-2-one**	**-627.44**	**-824.33 to -430.55**	**0.0017**	**0.0159**
**Cyclocitral**	**-73.49**	**-97.90 to -49.08**	**0.0020**	**0.0189**
**2,6-Nonadienal**	**30.82**	**19.74 to 41.91**	**0.0026**	**0.0244**
**Benzaldehyde**	**126.46**	**80.30 to 172.61**	**0.0027**	**0.0244**
**2-Octenal**	**1309.83**	**826.08 to 1793.57**	**0.0027**	**0.0244**
**Octanal**	**112.61**	**66.18 to 159.04**	**0.0037**	**0.0340**
**Phenylacetaldehyde**	**-28.24**	**-39.91 to -16.57**	**0.0037**	**0.0340**
**2-Nonenal**	**15.61**	**8.90 to 22.31**	**0.0041**	**0.0341**
2-Hexenal	34522.35	17219.15 to 51825.54	0.0071	0.0556
Decanal	94.17	42.26 to 146.07	0.0096	0.0714
3-Hexen-1-ol	640.86	281.83 to 999.88	0.0098	0.0714
3-Methoxy-1-butanol	256.72	102.04 to 411.39	0.0122	0.0784
1-Hexanol	28.00	10.61 to 45.39	0.0131	0.0784
Benzyl Alcohol	-38.32	-64.54 to -12.10	0.0177	0.0911
6-Methyl-3,5-heptadiene-2-one	-8.75	-14.91 to -2.59	0.0189	0.0911
Nonanal	185.89	21.29 to 350.49	0.0380	0.1390
Hexanoic acid	10.94	-0.69 to 22.57	0.0626	0.1772
3,7-Dimethyl-6-octen-1-oyl formate	27.98	-43.04 to 98.99	0.3453	0.6708
Acetic acid	0.44	-1.03 to 1.91	0.4499	0.6708
