# Supplementary figures and images for: Metabolic Responses of Grapevine Leaves to Grapevine Leafroll-Associated Virus 3 Infection
Source: Metabolites. 2026 May 27;16(6):359. doi: 10.3390/metabo16060359 (PMC13303152; doi:10.3390/metabo16060359)

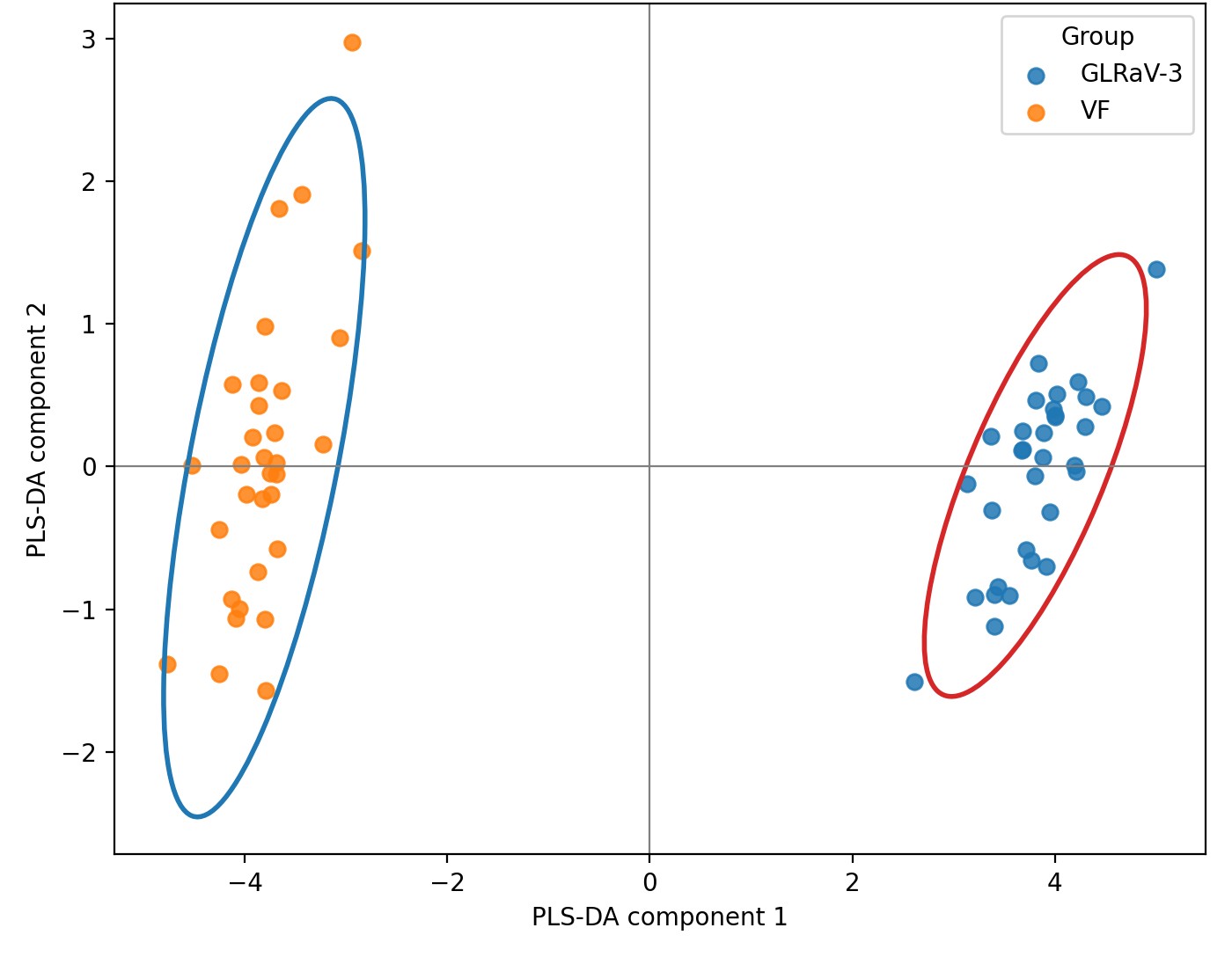

Supplement: Supplementary file 1 [file metabolites-16-00359-s001.zip › Supplemental Figure S1.jpeg]

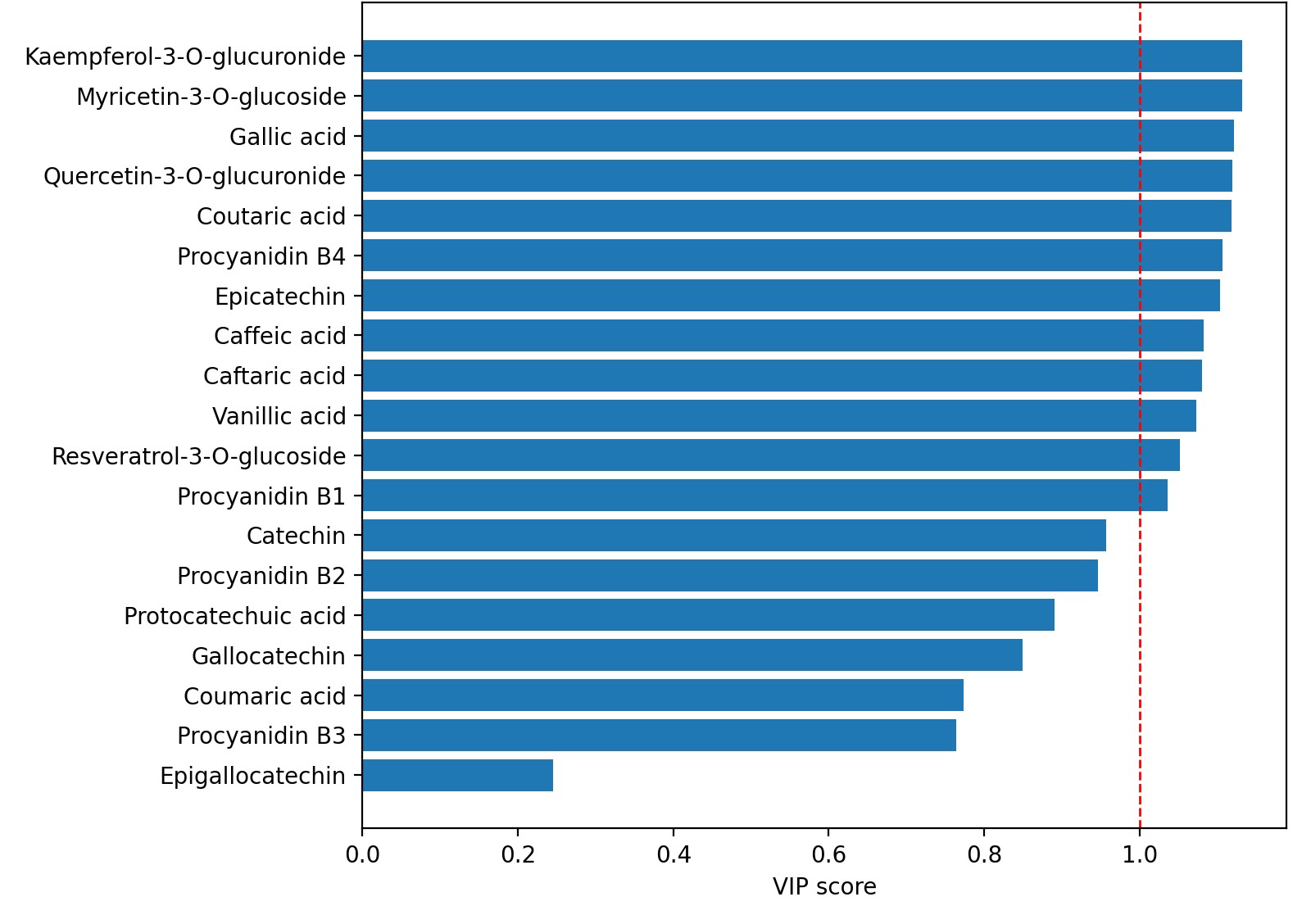

Supplement: Supplementary file 1 [file metabolites-16-00359-s001.zip › Supplemental Figure S2.jpeg]

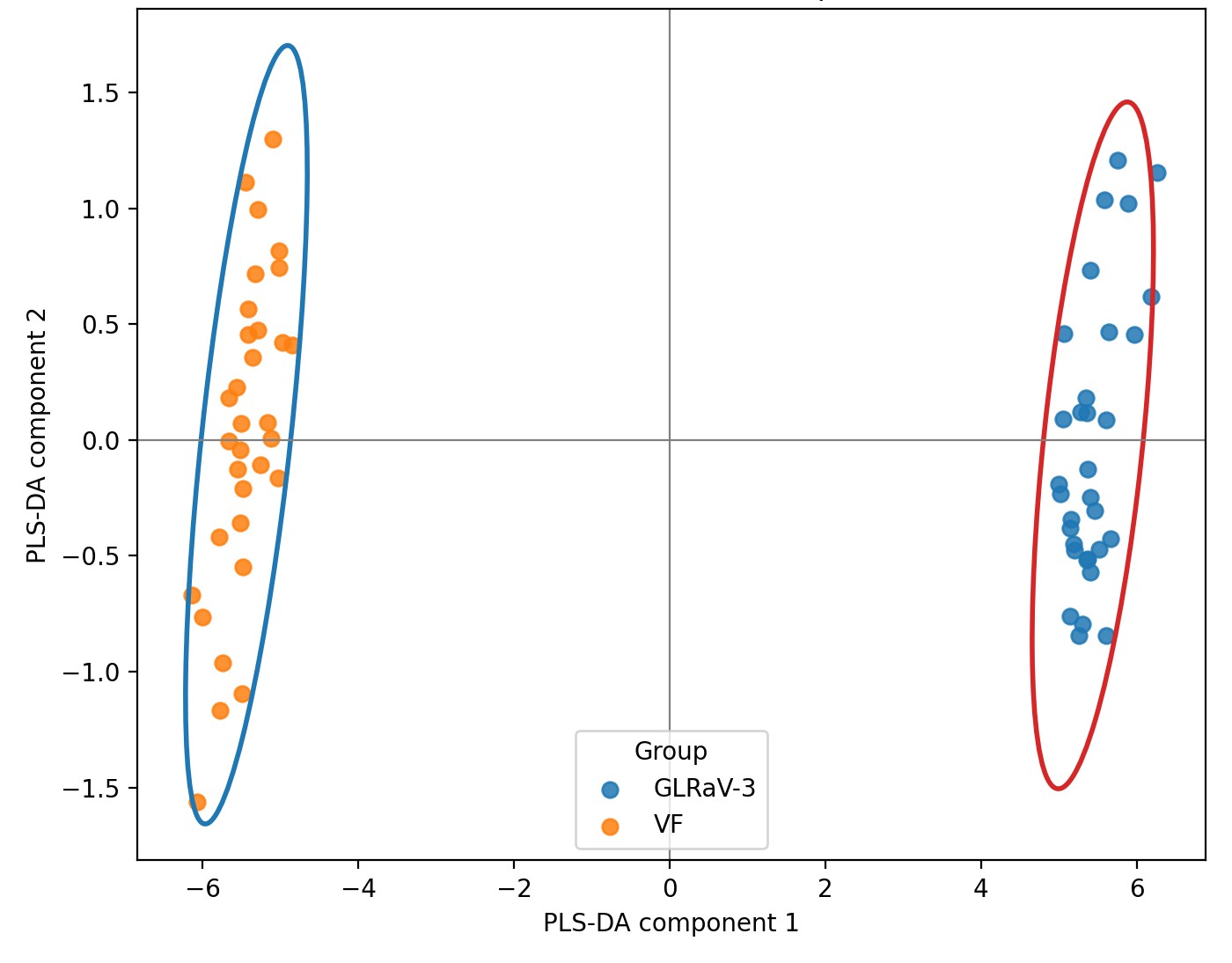

Supplement: Supplementary file 1 [file metabolites-16-00359-s001.zip › Supplemental Figure S3.jpeg]

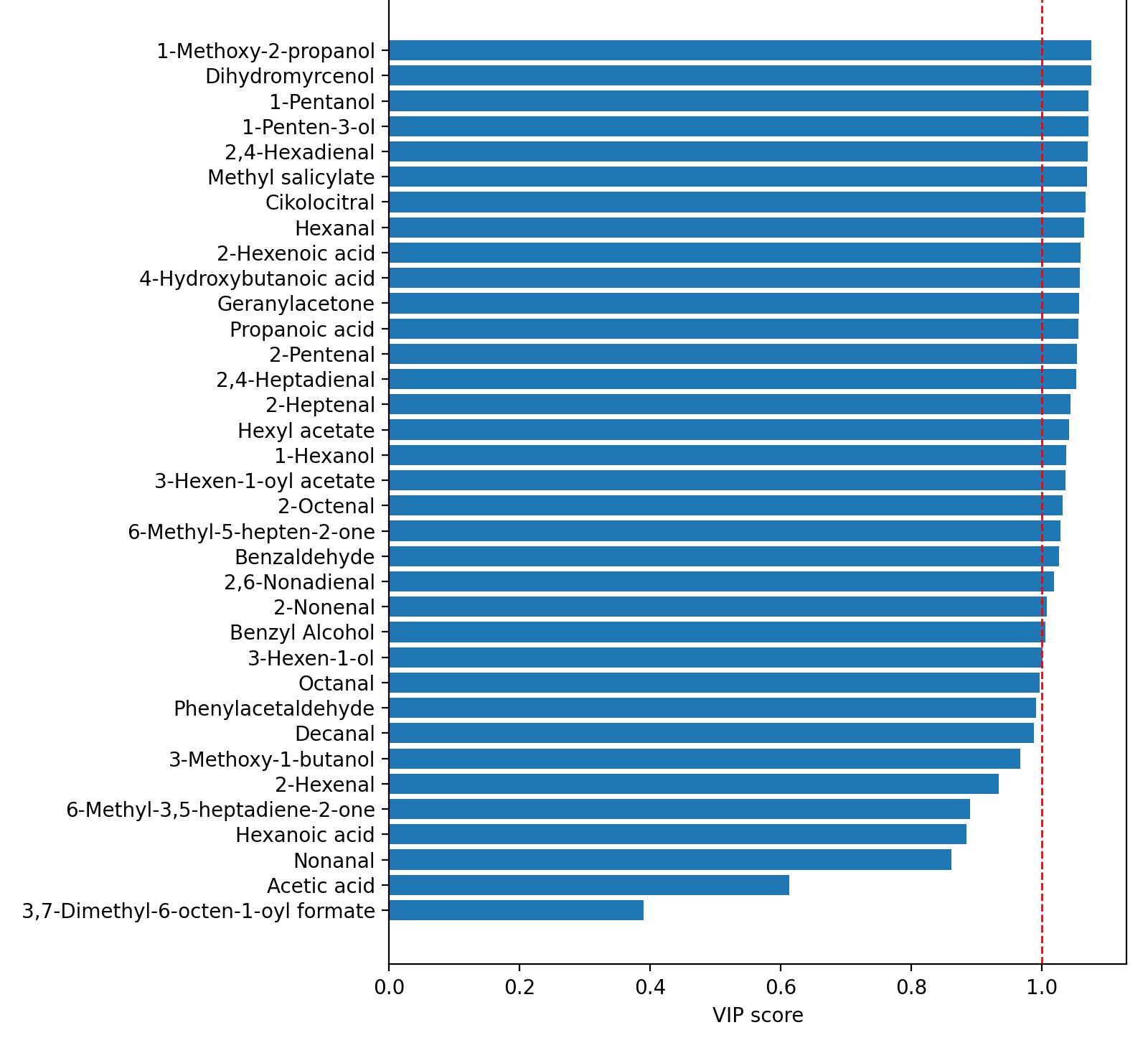

Supplement: Supplementary file 1 [file metabolites-16-00359-s001.zip › Supplemental Figure S4.jpeg]
